# Real-Time PCR: An Essential Guide

**DOI:** 10.3201/eid1101.040896

**Published:** 2005-01

**Authors:** Karen McCaustland

**Affiliations:** * Centers for Disease Control and Prevention, Atlanta, Georgia, USA

**Keywords:** Real-Time PCR: An Essential Guide, Book Review, Kirsten Edwards, Julie Logan, Nick Saunders, polymerase chain reaction

Real-time polymerase chain reaction (PCR) technique has advanced greatly over the past 10 years. This timely, comprehensive publication includes information on currently available instrumentation, fluorescent chemistries, assay design, optimization, and validation strategies. The background chapters are followed by chapters on quantification, single nucleotide polymorphism, mutation detection, and application of the various chemistries to clinical use in pathogen detection, gene expression, and human genetic testing. All of the chapters are well referenced; many of the contributing authors are recognized as respected experts in the field of real-time PCR.

Following a short overview of real-time PCR, the second chapter covers the various instruments currently on the market with a discussion on what features to look for when considering a purchase. The authors have put together a table of the machines, listing such details as the optics, the mode of detection (charge-coupled device camera or photomultiplier tube), the platform (96-well, glass capillary, etc.), and size and weight. The list contains every instrument except the latest offerings by ABI (7300 and 7500) and MJ (Chromo4). A couple of added features are given for some instruments but not others; for instance, the relative quantification software standard on the Stratagene Mx4000 and Mx3000p was not noted. Similar software is also available from ABI for their instruments. The list of websites after the references, containing general real-time PCR sites as well as those of the manufacturers and newsgroups, is an invaluable resource for both the novice and the veteran of real-time PCR.

Chapter 3 delves into the specific fluorescent chemistries, including intercalating dyes for generic detection of PCR product and template-specific designs such as linear hydrolysis (Taqman) and hybridization probes, and conformational probes (i.e., Molecular Beacons, Scorpions). One design not included was the Lux primers, a trademark design from Invitrogen. There is ample discussion on how the various chemistries work, design parameters, and examples of specific applications. The publisher might consider some changes in the placement of the tables and figures in this chapter. Most of the tables and figures are placed at the front of the chapter but not referred to until much later in the text, making it awkward for the reader to refer to them. Some attention to the font size and type for, which is hard to read, and the gray scale in many other figures throughout the book would improve the depiction of the illustrations.

The next 3 chapters (4–6) cover assay set-up and optimization, assay validation, and the design and use of controls for quantification. Chapter 4 is a general overview; chapter 5 discusses the use of internal and external controls, with the emphasis on the design and optimization of a synthetic mimic as an internal control. Chapter 6 deals with developing and using a quantitative standard. All 3 chapters address the importance of assay optimization and how this relates to PCR efficiency. They also stress the use of appropriate controls to identify false-positive results; more importantly, these chapters discuss how controls can identify false-negative results and their cause (PCR inhibitors, missing reagent components or test sample, or equipment problems), a must for diagnostic applications.

Chapter 7 deals with gene expression but I recommend that anyone considering real-time PCR read this chapter as a primer for what real-time PCR entails. The information on RNA extraction, reverse transcription, and amplification is extensive and includes discussion of the various enzymes, the master mixes and additives, and how they may or may not enhance recovery from any number of sources. The authors also cover optimization as it relates to reaction efficiency and relative versus absolute quantification. The monitoring of gene expression levels in response to viral load or cancer-producing tumor cells has become a critical part of treatment strategies, and the need for rapid, reliable assays has been effectively addressed with quantitative real-time PCR.

Comparison of how the different probe types (linear hybridization, hydrolysis, and conformational) and the Scorpion-labeled primer work in mutation detection; SNPs are covered in chapter 8. Examples are given for how real-time PCR detection can be applied to identify human genetic diseases (Factor V Leiden and cystic fibrosis, for example) or to diagnose drug-resistant bacteria for proper drug therapy. The design principles and optimization strategies are demonstrated in a specific application to detect low levels of a lamivudine–resistant hepatitis B mutant and its use in drug therapy management. Another unique real-time assay, nucleic acid sequence–based amplification (NASBA), is discussed in chapter 10. These isothermic NASBA assays are used most often for RNA detection and or quantification. Reactions require avian myeloblastosis virus reverse transcriptase, ribonuclease H, T7 RNA polymerase in the master mix, and a thermostatic fluorimeter, the probe of choice is the Molecular Beacon.

The final 2 chapters cover a myriad of applications used in clinical microbiology and the diagnosis of infectious diseases. Even though presented as an overview, the >100 references in chapter 11 illustrate how vast and varied the application of real-time PCR, and the technological advances to support its use, have become in the past decade. This publication would be a good addition to any laboratory as an up-to-date resource for both the novice and the experienced researcher.

